# Radiomics and Radiogenomics in Pelvic Oncology: Current Applications and Future Directions

**DOI:** 10.3390/curroncol30050372

**Published:** 2023-05-11

**Authors:** Niall J. O’Sullivan, Michael E. Kelly

**Affiliations:** 1 The Trinity St. James’s Cancer Institute, D08 NHY1 Dublin, Ireland; 2 School of Medicine, Trinity College Dublin, D02 PN40 Dublin, Ireland

**Keywords:** radiomics, radiogenomics, oncology, survival, recurrence, treatment response

## Abstract

Radiomics refers to the conversion of medical imaging into high-throughput, quantifiable data in order to analyse disease patterns, guide prognosis and aid decision making. Radiogenomics is an extension of radiomics that combines conventional radiomics techniques with molecular analysis in the form of genomic and transcriptomic data, serving as an alternative to costly, labour-intensive genetic testing. Data on radiomics and radiogenomics in the field of pelvic oncology remain novel concepts in the literature. We aim to perform an up-to-date analysis of current applications of radiomics and radiogenomics in the field of pelvic oncology, particularly focusing on the prediction of survival, recurrence and treatment response. Several studies have applied these concepts to colorectal, urological, gynaecological and sarcomatous diseases, with individual efficacy yet poor reproducibility. This article highlights the current applications of radiomics and radiogenomics in pelvic oncology, as well as the current limitations and future directions. Despite a rapid increase in publications investigating the use of radiomics and radiogenomics in pelvic oncology, the current evidence is limited by poor reproducibility and small datasets. In the era of personalised medicine, this novel field of research has significant potential, particularly for predicting prognosis and guiding therapeutic decisions. Future research may provide fundamental data on how we treat this cohort of patients, with the aim of reducing the exposure of high-risk patients to highly morbid procedures.

## 1. Background

Radiomics refers to the conversion of medical imaging into high-throughput, quantifiable data in order to analyse disease patterns, guide prognosis and aid in decision making [1,2]. Since its inception in 2012, data have been extracted and analysed from a variety of imaging modalities, such as computed tomography (CT), magnetic resonance imaging (MRI) and positron emission tomography (PET) scans, in an attempt to determine prognosis and to predict patient outcomes, particularly in the field of surgical oncology [1,3]. The process can be broadly broken down into three primary steps: the use of automated or semi-automated methods to identify the volumes of interest in a tumour, the generation of multiple quantitative features from raw imaging data regarding the region of interest (ROI), and finally the development of models that predict tumour characteristics and guide decision making [4].

Radiogenomics is an extension of radiomics that combines conventional radiomics techniques with molecular analysis in the form of genomic and transcriptomic data [5]. This particular field of medicine aims to offset the high costs and labour intensity associated with traditional genetic testing via the development of imaging surrogates that may serve as an alternative method for identifying which patients carry specific oncogenes [6]. In short, parameters derived from advanced image processing and analysis can be used to identify specific phenotypic and genotypic characteristics of the tumour, without the need for costly genetic testing [7]. The ultimate goal of radiogenomics is to offset the high costs, workload and invasiveness associated with traditional genetic testing by developing imaging surrogates that have the potential to serve as alternative methods for identifying oncogene status [6]. Its role has been assessed in several fields of oncology, including glioblastoma, breast cancer, renal cell carcinoma and colorectal cancer [8,9,10]. While several studies have demonstrated the feasibility of constructing a radiogenomic signature, most lack prospective validation with an external cohort [11,12].

Artificial intelligence (AI) is increasingly employed in medicine due to its ability to perform higher cognitive functions, such as problem solving and decision making [13]. A subfield within AI, machine learning facilitates the ability to search for data and recognize patterns, allowing the accurate prediction of results or outcomes [14,15]. This is particularly useful for identifying subtle patterns in large datasets that are often imperceptible to individual review [13,16]. The incorporation of machine learning with other new fields of research, such as radiomics and radiogenomics, has the potential to revolutionise precision medicine by predicting treatment response, prognosis and patient outcomes [17].

**Figure 1:**
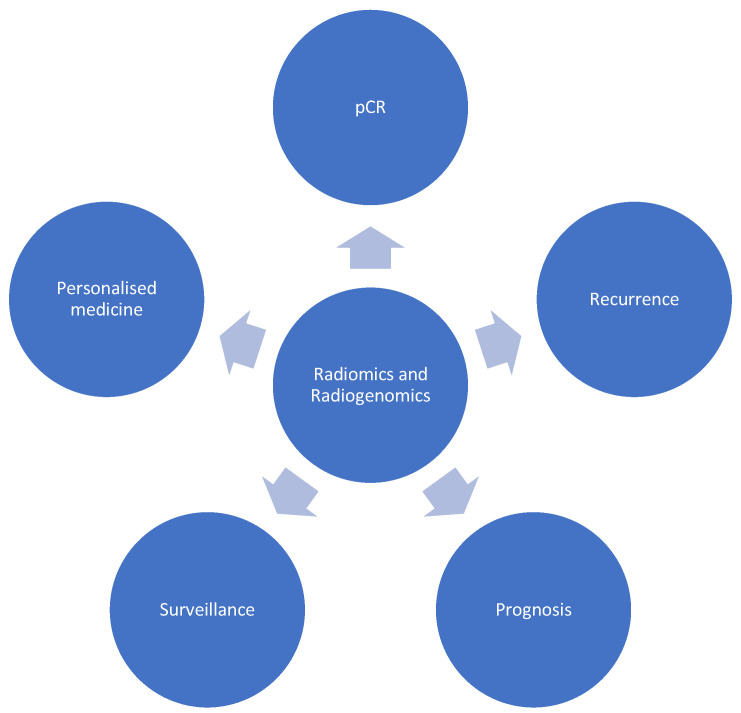
Applications of radiomics and radiogenomics in pelvic oncology. pCR: Pathological complete response.

This review aims to shed light on the current implementation of radiomics and radiogenomics in the field of pelvic oncology, paying particular attention to individual subspecialty use and implications for the future. We provide a sample of the current literature within this field, highlighting the strengths, weaknesses and gaps in knowledge that may guide researchers in their future endeavours. Included studies deemed interesting and representative of the literature were selected after relevant articles were searched in the National Institutes of Health (NIH) PubMed database. Broad applications are highlighted in Figure 1.

## 2. Current Applications of Radiomics and Radiogenomics

### 2.1. Colorectal

(i) Prediction of response to neoadjuvant chemotherapy

Several studies have investigated the application of radiomics and radiogenomics in colorectal surgery [18,19,20,21,22,23,24]. Nakanishi et al. developed a radiomics-based model for predicting lateral pelvic lymph node metastases (LPLNM) following neoadjuvant (chemo)radiotherapy (nCRT) for advanced low rectal cancers [24]. A retrospective study of 247 patients demonstrated that a radiomics-based prediction model was superior to the gross measurement of LPLN short-axis diameter in the prediction of LPLNM post-neoadjuvant (chemo)radiotherapy for rectal cancer [24]. This study demonstrated how a radiomics-based prediction model can be applied to avoid unnecessary exposure of patients to high morbidity procedures such as LPLN dissection in those patients deemed ‘low risk’ by the model. Recent studies have investigated the feasibility of radiomics nomograms in predicting a pathological complete response (pCR) [25,26]. Liu et al. developed a radiomics-based model using pre- and post-nCRT T2 and diffusion-weighted images (DWI) in combination with tumour length, with the ability to predict pCR with a diagnostic accuracy of 94% [25]. Similarly, Wang et al. extracted radiomic features from 183 pre-operative mpMRI scans to develop a model capable of predicting the response to nCRT [26]. Their nomogram, which included MRI T-stage, circumferential resection margin and apparent diffusion coefficient values, predicted a good response to nCRT with a specificity of 88% and a sensitivity of 71% [26].

(ii) Prediction of mutation status

The role of fluorodeoxyglucose (^18^F-FDG) PET in the assessment of KRAS mutations in colorectal cancer is another topic of research in colorectal radiogenomics [18]. This radiotracer detects areas of abnormal glucose metabolism and serves to evaluate metabolic and tumour activity [27]. Several studies have demonstrated a higher standardised uptake value (SUV) of this radiotracer in patients with KRAS mutations, with a reported diagnostic accuracy of up to 75% [28,29]. MRI remains the gold standard imaging modality for rectal cancer due to its ability to assess extramural spread, accurately stage and detect local recurrence of the disease [30]. Studies have demonstrated that rectal tumours with KRAS mutations are more likely to exhibit a longer axial length as well as a greater axial: longitudinal ratio on pre-treatment MRI [31,32]. Since genomic analysis is now essential to guide therapeutic decisions in colorectal cancer, the development of radiogenomic models capable of predicting the involvement of various genetic mutations will allow for targeted therapy and improved patient outcomes [18]. Radiogenomic prediction of specific genetic aberrations has the potential to serve as a non-invasive alternative to conventional genetic testing in the future [33]. Further studies should focus on applying this novel technology to other genetic mutations as well as validating the findings of existing studies.

(iii) Prediction of oncological outcomes

Exenterative surgery can be associated with major complications [5]. Despite advancements in surgical techniques and our understanding of advanced and recurrent pelvic malignancies, the rate of recurrence after pelvic exenteration remains unacceptably high [34]. Radiomics and radiogenomics offer the potential to offset the risk of recurrence in this cohort of patients by accurately predicting which patients are likely to experience recurrence and subsequently avoid exenterative surgery and its associated morbidity [3,35]. Badic et al. aimed to use radiomics to assess the value of contrast-enhanced CT scans as predictors of recurrence in patients with stage II and III colorectal cancer [36]. The authors used three separate machine learning models to predict disease-free survival (DFS) in this cohort of patients. A signature was developed based on clinical, histopathological and radiomic characteristics, and a predictor of recurrence showed value when compared with the traditional staging [36]. Similarly, Jayaprakasam et al. demonstrated the capabilities of MRI radiomics in predicting tumour recurrence and response to neoadjuvant chemotherapy in patients with locally advanced rectal cancer [37]. Their study exploited the hypothesis that the tumour and mesorectal fat interaction results in microscopic changes to adipocytes and subtle changes on MRI that are invisible to the naked eye [37]. In terms of predicting response, eleven radiomics features differed significantly between complete and non-complete responders. The final predictive model obtained a diagnostic accuracy of 83.9% and an area under the curve (AUC) of 0.89. Univariate analysis revealed 36 radiomics features that were significant in predicting local recurrence. The final predictive model obtained a diagnostic accuracy of 78.3% and an AUC of 0.79 [37]. These studies demonstrated the feasibility and efficacy of employing radiomics models in pelvic malignancies to predict various oncological outcomes such as response to chemotherapy and risk of recurrence, with relatively strong accuracy.

### 2.2. Urological

(i) Prostate cancer; prediction of oncological outcomes

Recent years have seen a rapid increase in the number of publications applying radiomics and radiogenomics to genitourinary cancers [38]. In the realm of prostate cancer, Bourbonne et al. developed a multiparametric MRI (mpMRI)-based radiomic model to predict the risk of biochemical recurrence (BCR) and BCR-free survival post-radical prostatectomy [39]. The authors used a size-zone emphasis from apparent diffusion coefficient (ADC) maps extracted from 107 pre-therapeutic diffusion-weighted images, and demonstrated a balanced accuracy of 78% in predicting BCR post-radical prostatectomy [39]. This model was externally validated and may be used to stratify patients post-operatively by the risk of recurrence and tailor post-operative management accordingly. External validation is a labour-intensive but valuable component of radiomic nomogram construction, verifying reproducibility across other centres. The Miami MAST trial (ClinicalTrials.gov: NCT02242773), currently ongoing, aims to extract data from mpMRI-guided MRI/ultrasound (US) fusion biopsies in order to identify high-grade tumours early on in the investigations [40]. This study is estimated to be completed in 2024 and will aid physicians in their decision to commence radical treatment earlier in high-risk diseases. The current literature on radiogenomics in prostate cancer is limited, particularly regarding the detection of specific biomarkers that may be used to guide prognosis and treatment decisions [41]. The three current commercially approved genomic panels performed on prostate biopsy cores (Genomic Health’s Oncotype Dx test^®®^, Myriad’s Prolaris test^®®^ and Genome Dx’s Decipher test^®®^) will provide a good basis for future research into prostate cancer radiogenomics [42,43,44].

(ii) Bladder cancer; prediction of oncological outcomes

Several studies have investigated the role of radiomics and radiogenomics in predicting clinical outcomes in bladder cancer [45,46,47,48]. Lin et al. sequestered RNA sequencing data, radiomics features and clinical parameters of 62 patients with transitional cell carcinoma (TCC) of the bladder to create an integrative nomogram capable of stratifying patients into low- and high-risk groups and subsequently predicting progression-free interval (PFI) with excellent accuracy [46]. CD8A is a novel protective gene in bladder cancer and a marker of immunotherapeutic response and immune cell infiltration [48]. Low expression is associated with immunotherapeutic failure and poor oncological outcomes [48]. Zheng et al. developed a radiomics signature using pre-operative radiomics features and RNA-sequencing data of 111 bladder tumour samples to predict CD8A expression and subsequent response to immunotherapy [48]. Receiver operating characteristic (ROC) curves revealed that the nomogram had good performance in survival prediction with 1-, 3- and 5-year area AUC of 0.679, 0.722 and 0.722, respectively. This study demonstrated the ability of a radiomics signature based on nine MRI-derived radiomics features to predict the prognosis and immunotherapeutic susceptibility in patients with bladder cancer [48]. While studies aiming to predict muscle invasiveness of bladder cancer are bountiful, those aiming to construct nomograms capable of predicting oncological outcomes are scarce [49,50,51]. Future research should focus on predicting the response to neoadjuvant therapy and the long-term oncological outcomes.

### 2.3. Gynaecological

(i) Ovarian cancer; prediction of BRCA status

Despite recent progress in chemotherapeutic and surgical approaches, the high morbidity and mortality associated with many gynaecological malignancies necessitate an improved understanding of how these tumours behave from a radiological and genetic perspective [52]. Few studies have described the use of radiomics and radiogenomics in gynae-oncology [52,53,54,55]. Nero et al. developed an automated machine learning pipeline model in order to identify gBRCA1/2 status based on ultrasound images of healthy ovaries, with encouraging performance [54]. With a positive predictive value (PPV) of 0.87, only 13/100 women will be exposed to unnecessary genetic testing. Despite this, the model describes a negative predictive value (NPV) of 0.67, implying that 27/100 women carrying the gene would be missed; thus, it is a major limitation of the approach [54].

(ii) Endometrial cancer; prediction of oncological outcomes

Hoivik et al. integrated MRI radiomic features with histologic, transcriptomic and molecular biomarkers in their study of 866 patients with endometrial cancer in an attempt to identify those with aggressive tumour features [55]. In this study, a fully automated machine learning-based tumour segmentation algorithm reproduced the same radiomic prognostic groups as manual whole-volume tumour radiomic profiling by radiologists [55]. The authors identified an 11-gene high-risk signature associated with poor survival that will aid in prognosis and guide treatment decisions in patients with endometrial carcinoma. Further research is necessary to bring radiomics-based research within gynae-oncology to the same standard as currently available in the fields of colorectal and urology.

### 2.4. Sarcoma

The use of artificial intelligence in soft-tissue sarcomas is a relatively novel concept that aims to serve as a non-invasive method of providing information regarding the diagnosis and prognosis of tumours [56]. The employment of radiomic texture analysis in this field has resulted in the development of radiomics MRI-based models that can distinguish histotypes, determine grades and predict response and overall survival [56]. Using T1 and fat-suppressed-T2 weighted imaging, Wang et al. identified specific radiomic features that were significantly correlated with malignant soft-tissue lesions and subsequently constructed a radiomics nomogram with superior predictive performance than that of the clinical model based on the experience of radiologists [56]. Peeken et al. compared the value of MRI-based radiomics with expert-derived clinical profiling for the prediction of overall survival (OS) [57]. A total of 105 radiomic features were extracted from the images of 108 patients and subjected to three separate machine learning techniques to predict OS. The findings were compared to the semantic imaging features determined by radiologists. T2-weighted sequence and T1-weighted fat-saturated sequence radiomic models were superior to semantic imaging features in determining the prognosis of soft-tissue sarcomas [57]. To our knowledge, no studies investigating the use of radiomics or radiogenomics in the diagnosis and prognosis of intra-abdominal sarcomatous disease have been published. Future studies should focus on filling this void, paying particular focus to the prediction of oncological outcomes in patients with this aggressive disease.

## 3. Current Limitations

Despite significant potential in the field of pelvic oncology, current evidence is limited by variability in feature extraction and a lack of reproducibility [58,59]. Model performance is sensitive to many intrinsic variables, including heterogeneous image acquisition parameters, segmentations and feature extraction software, as well as small and mixed patient cohorts [60]. As research in this field advances, more open-source databases and software packages are being made available in an attempt to standardise models and accelerate the development and external validation of these signatures [61,62,63]. Future studies should focus on standardising the imaging protocols and radiomic techniques [18]. Standards for radiomic features need to be set in order to allow comparability between studies and reproducibility for new studies. Similarly, the stability and reproducibility of radiomic models for predicting prognosis must be externally assessed prior to their application in the clinical setting [64]. Internal validation may not be sufficient to extrapolate performance in an external setting due to relatively small datasets, as seen in most of the current studies, and external datasets should thus be validated in a large multicentre setting prior to implementation in clinical practice [65].

Multiple studies have attempted to identify reproducible radiomics features to improve the repeatability and application of radiomics and radiogenomics. Traverso et al. performed a systematic review to identify radiomics features that were repeatable and reproducible [66]. The authors found that first-order features (histogram-based), in particular entropy, had higher reproducibility than shape metrics and textural features. The Image Biomarker Standardisation Initiative (IBSI), published in 2020, aimed to standardise a set of radiomics features [67]. Over three phases, the authors achieved good to excellent reproducibility for 167 different radiomics features utilising CT, MRI and FDG-PET in 51 patients with soft-tissue sarcoma. Similarly, Pfaehler et al. developed a checklist aiming to simplify and improve the reporting of radiomic signatures, with the goal of eventually guaranteeing full replication and validation of these studies [68]. Further systematic reviews focusing on the repeatability and reproducibility of radiomics features are necessary to further improve the standardisation of results from radiomics studies [69].

## 4. Future Directions

The use of radiomics and radiogenomics for predicting correlations with genetic or transcriptomic abnormalities of tumours and subsequently determining prognosis and guiding treatment decisions needs much larger data studies to become a validated tool [18]. Future studies should focus on addressing the limitations outlined previously and evaluating the datasets in a multicentre, prospective setting. There is a paucity of evidence in the literature surrounding the use of radiomics and radiogenomics to identify patients at high risk of recurrence of locally advanced or locally recurrent pelvic cancers. The development of a radiomics nomogram aimed at predicting disease recurrence in this cohort of patients may offer surgeons the ability to avoid highly morbid procedures, such as exenteration in patients who are likely to recur, or at least counsel patients better regarding expected outcomes [5].

Quantification of circulating tumour DNA (ctDNA) and cell-free DNA (cfDNA) represents a novel area of research in cancer detection and evaluation of disease burden, with the potential to revolutionise our assessment of therapeutic responses and our understanding of personalised medicine as a whole [70]. Current applications include the detection of microscopic residual disease following radiotherapy, an alternative non-invasive method of genotyping, as well as early detection of tumour recurrence [71]. Research on ctDNA in the field of radiomics and radiogenomics is scarce [72]. Lafata et al. aimed to create patient-specific radiogenomic expression patterns to guide prognosis by combining CT radiomics, next-generation sequencing of ctDNA and serum cfDNA in patients with locally advanced lung cancer receiving chemoradiotherapy [72]. Two distinct radiomic signatures were identified prior to treatment, which were subsequently associated with the presence of TP53 mutations within ctDNA and changes in cfDNA two weeks post-chemoradiation. The authors found that heterogeneous and low-attenuating disease, without a detectable ctDNA TP53 mutation, was linked to an early surge in post-treatment cfDNA concentration and improved overall survival [72]. These findings highlight the feasibility and efficacy of this approach in predicting treatment response and guiding prognosis. Further studies, ideally larger randomised clinical studies, are necessary to validate these findings.

Perhaps one of the greatest obstacles to radiomic-based research is feature reliability [73]. Heterogeneity and uncertainty can arise from many areas within a complex workflow, ultimately impeding feature reproducibility, stability and validity [74,75,76,77,78,79]. In their review of 481 radiomics studies, Xue et al. attempted to define reliability using intraclass correlation coefficient (ICC) expression [73]. ICC is a reliability index widely adopted in the medical literature, which can be applied to any radiomic feature that has continuous values [73,80]. The authors conclude by offering several suggestions for researchers carrying out radiomics-based research to mitigate the pitfalls identified in their analysis of 481 manuscripts. Koo et al. also provide clinical researchers with a practical step-by-step guideline to select the correct form of ICC and to avoid selecting inappropriate ICC forms that may result in misleading interpretations [80].

## 5. Conclusions

Despite a rapid increase in publications investigating the use of radiomics and radiogenomics in pelvic oncology, current evidence is limited by poor reproducibility and small datasets [60]. In the era of personalised medicine, this novel field of research has significant potential, particularly for predicting prognosis and guiding therapeutic decisions [3,76]. Future research may provide fundamental data on how we treat this cohort of patients, with the aim of reducing the exposure of high-risk patients to highly morbid procedures. While many of the studies discussed in our narrative review are small, single-centre projects, they provide the foundation necessary and proof of efficacy required for the production of further high-impact studies in the future. Large-scale, multi-institutional studies with external validation of models are required to ultimately change clinical practice.

## Data Availability

No new data was created in this review.

## Abbreviations

CT: Computed Tomography, MRI: Magnetic Resonance Imaging, PET: Positron Emission Tomography, ROI: Region of Interest, AI: Artificial Intelligence, LPLNM: Lateral Pelvic Lymph Node Metastasis, nCRT: Neoadjuvant Chemoradiotherapy, pCR: Pathological Complete Response, SUV: Standard Uptake Value, FDG: Fluorodeoxyglucose, DFS: Disease-free survival.
